# Electroacupuncture-Induced Dynamic Processes of Gene Expression Levels of Endogenous Opioid Peptide Precursors and Opioid Receptors in the CNS of Goats

**DOI:** 10.1155/2013/257682

**Published:** 2013-05-16

**Authors:** Li-Li Cheng, Ming-Xing Ding, Jia Wei, Yi-Qiang Wu, Zheng-Ying Qiu, Jian-Guo Chen, Dong-Ming Liu, Chang-Min Hu, Man-Li Hu, Zahir Shah, Qiong Wang

**Affiliations:** College of Veterinary Medicine, Huazhong Agricultural University, Wuhan 430070, China

## Abstract

In order to investigate the dynamic processes of mRNA levels of proenkephalin, proopiomelanocortin, prodynorphin, and opioid receptors (*δ*-, *μ*-, and *κ*-receptor) induced by electroacupuncture (EA) in the central nerve system, goats were stimulated by EA of 60 Hz for 0.5 h at a set of Baihui, Santai, Ergen, and Sanyangluo points. The pain threshold was measured using the method of potassium iontophoresis. The mRNA levels of the three opioid peptide precursors and three opioid receptors were determined with quantitative real-time PCR and the levels of Met-enkephalin with SABC immunohistochemistry at 0.5 h before and at 0, 2, 4, 6, 8, 12, and 24 h after EA. The results showed that the pain threshold correlated (*P* < 0.01) with Met-enkephalin immunoactivities in the measured nuclei and areas of goats. The analgesic aftereffect lasted for 12 h at least. The mRNA levels of the three opioid peptide precursors and three opioid receptors began to increase at 0 h, reached the peak during the time from 4 h to 6 h or at 12 h, and remained higher at 24 h after EA was discontinued. These results suggested that the initiation of gene expression of opioid peptides and the three receptors may be associated with EA-induced analgesic aftereffect.

## 1. Introduction

Electroacupuncture (EA) is a modern version of acupuncture and extensively used in the clinic practice because it has better analgesic effect and its stimulation can be objectively quantified and controlled [1]. EA has been used not only for effective treatment of painful diseases, but also for successful relief of pain in various operations, such as cesarean section, gastrectomy, enterectomy, and castration in human or animals [2–4]. Since the 1960s, many scientists have investigated the mechanism by which electroacupuncture induces analgesic effect. Early studies showed that analgesia induced by EA was involved in modulations of neurotransmitters (serotonin, acetylcholine, catecholamine, etc.) in the central nerve system (CNS) [5]. Latter, studies verified that neuromodulators, especially some endogenous opioid peptides (enkephalin, *β*-endorphin, and dynorphin), played a more important role in EA-induced analgesia. EA can promote the release of different endogenous opioid peptides (EOPs) which act on their corresponding receptors (*δ*-, *μ*-, or *κ*-receptor) to exert analgesic effect [6–9].

 Previous studies indicated that EA not only induces an “immediate analgesia,” but also causes an analgesic aftereffect (analgesia lasts for a while after EA is discontinued). This aftereffect plays an important role in the treatment of painful diseases and is conducive to the recovery from the surgery. So far the mechanisms by which acupuncture induces analgesic aftereffect have not been fully studied. Some reports showed that EA induced the gene expression of opioid peptide precursors, such as proopiomelanocortin (POMC, precursor of endorphin), proenkephalin (PENK, precursor of enkephalin), and prodynorphin (PDYN, precursor of dynorphin) in the CNS of rats [10–12]. The initiation of EOP gene expressions is inferred to replenish the consumed opioid peptides because opioid peptides are immediately decomposed by some specific enzymes after they are induced to release and act on their corresponding receptors. However, the roles of the gene expressions of endogenous opioid peptides and their receptors in EA-induced aftereffect are not completely confirmed. 

 Analgesia induced by EA has been proven to vary in animal species [1]. In order to quantitatively estimate the degree of acupuncture-induced analgesia, some researchers compared the dosage of some anesthetic in the anesthetic group with its dosage in the EA plus anesthetic group with the same complete analgesia. They found that EA resulted in the reduction of the dosage of anesthetics in the EA plus anesthetic group in human, rat, and goat by 45%–55%, 50%–60%, and over 75%, respectively [13, 14]. It is clear that the analgesic effect induced by EA in goats (a ruminant) is superior to that in rats or human. Ruminants should be optimal model animals for researches on the mechanisms of EA-induced analgesia. In the present study, goats were stimulated with EA for 30 min to determine the relationships of the gene expression of Met-enkephalin (M-ENK), beta-endorphin (*β*-EP), and dynorphin (DYN-A) and their receptors (*δ*-, *μ*-, and *κ*-receptor) with pain threshold and levels of enkephalin (a representative of opioid peptides) in order to probe into the mechanisms of EA-induced aftereffect. 

## 2. Materials and Methods

### 2.1. Animal Preparation

 One hundred and eight healthy 1- to 2-year-old hybrid male goats, weighing 23–28 kg, purchased from the goat farm of Hubei Agricultural Academy of Science, were used in this experiment (54 goats for the measurement of gene expression levels of endogenous opioid peptides and opioid receptors, another 54 goats for the measurement of M-ENK levels). All goats drunk freely and were maintained on a dry grass diet which was supplemented with a cereal-based concentrate. They were dewormed and accustomed to being approached. Feed was withheld for 24 h before the start of the experiments. The experiments were performed in a quiet environment, and the ambient temperature fluctuated between 23°C and 24°C. The experimental protocol was approved by the Animal Care Center, College of Veterinary Medicine, Huazhong Agricultural University, Wuhan, China.

### 2.2. Electroacupuncture

A set of Baihui (hundred meetings), Santai (three platforms), Ergen (ear base), and Sanyangluo (three Yang communication) points was selected for EA. The anatomic location of these points has been described in detail in veterinary medicine [13, 15]. The Baihui and Santai points on the dorsal midline and the Ergen and Sanyangluo points on the left side of the body were chosen in this study. Needle insertion and EA were conducted with the method reported by Liu et al. [13]. Experimental animals were restrained in right recumbency and stimulated with EA at 60 Hz for 0.5 h via WQ-6F Electronic Acupunctoscope (Beijing Xindonghua Electronic Instrument Co., Ltd., Beijing, China). The sham control goats which were only dealt with needles left in the acupoints for 0.5 h without electricity were restrained as the experimental goats. 

### 2.3. Determination of Pain Threshold

At 0.5 h before and at 0, 2, 4, 6, 8, 12, and 24 h after EA, the pain threshold was measured on the center of the left flank using the method of potassium iontophoresis as described in detail by Cheng et al. [16]. The pain threshold in the sham control was measured at 0.5 h before needle insertion and at 0 h after needle withdrawal. 

### 2.4. Measurement of Gene Expression Levels of Endogenous Opioid Peptides and Opioid Receptors

Six goats were taken from the experimental goats at 0.5 h before and at 0, 2, 4, 6, 8, 12, and 24 h after EA, respectively, anesthetized with intravenous administration of xylidinothiazoline at 3 mg/kg, and slaughtered for the measurement of gene expression levels of endogenous opioid peptides and opioid receptors. According to the results of repeated pretest, gene expression of endogenous opioid peptides and opioid receptors in most nuclei and areas in the CNS reached a higher level at about 4 h after EA. Therefore, six sham control goats in the present study were euthanized for brain sampling at 4 h after needle withdrawal. The goat's brain, hypophysis, and a part of the adjacent spinal cord were immediately taken out of the skull and cervical vertebral canal. The brain was transected into 17 sections quickly with the method described by Cheng et al. [16]. The nuclei and areas were identified according to the photographic atlas of the goat brain and the morphological characteristics of the neurons [17–19]. The analgesia-related nuclei and areas were obtained with 4–8 mm diameter plastic tubes dealt with 1%  DEPC solution and then put into RNAstore solution (Beijing Tiangen Biological Technology Ltd., Beijing, China) to prevent RNA degradation. The mRNA levels of EOP precursors and the three opioid receptors were examined in the nuclei or areas in bilateral brain regions of goats.

 The gene expression levels of PENK, POMC, PDYN, and opioid receptors (*δ*-, *μ*-, and *κ*-receptor) were measured through the method of quantitative real-time PCR using ABI Prism 7500 real-time PCR instrument (ABI Co., USA). The PCR primers were designed according to the sequences of *β*-actin-mRNA (GeneBank accession no. AF481159), PENK-mRNA (GeneBank accession no. NM174141), POMC-mRNA (GeneBank accession no. NM001009266), PDYN-mRNA (GeneBank accession no. NM174139), *δ*-receptor-mRNA (GeneBank accession no. NM001191148), *μ*-receptor-mRNA (GeneBank accession no. AF266480), and *κ*-receptor-mRNA (GeneBank accession no. DQ065757). The PCR products were tested through the method of normal PCR. The homology between the referenced sequences and the products' sequences was greater than or equal to 95%. The sequences of PCR products had been submitted to GeneBank (accession nos. GU169095 for PENK, GU167924 for POMC, GU169905 for PDYN, JQ756319 for *δ*-receptor, JQ241177 for *μ*-receptor, and JQ241178 for *κ*-receptor). Their upstream and downstream primers were presented in Table 1. All experimental data were analyzed using the method of 2^−ΔΔCt^ through SDSShell software (ABI Co., USA).

### 2.5. Measurement of M-ENK Level

 The level of M-ENK was measured through the method of SABC immunohistochemistry. The experimental goats were taken and anesthetized as above. According to the previous research results that opioid peptides in the CNS were released to the higher level at the end of EA [16], six sham control goats in this study were anesthetized and perfused immediately after needle withdrawal. The goats were fixed with the infusion of 4% paraformaldehyde through bilateral carotid arteries. The goat's brain was taken out and transected into 17 sections with the method described by Cheng et al. [16]. Each of the subsections was embedded in a paraffin block, sectioned at 5 *μ*m, mounted on polylysine-coated slides, deparaffined, and rehydrated sequentially. Four serial slides were chosen from near the middle of each section for immunohistochemical staining. Of these four slides, the three were incubated with rabbit-anti-M-ENK IgG (1 : 100) (Wuhan Boster Biological Technology Ltd., Wuhan, China), while the rest was incubated with PBS instead of the corresponding antibody as negative control. Experimental procedures of SABC immunohistochemistry followed the instructions provided by the reagent company (Wuhan Boster Biological Technology Ltd., Wuhan, China). The cytoplasm of positive cells was stained as brown yellow. Optical density of the stained nuclei or areas in the CNS was obtained with a light microscope connected to a video-based and computer-linked system (high-resolution pathological image analysis system-1000, Wuhan Qianping Ltd., Wuhan, China). This system was programmed to calculate the mean optical density (MOD) for three fields of each slide examined under 400x magnification. The level of M-ENK in each nucleus or area was represented with the mean value %  of the mean optical density from the three slides.

### 2.6. Statistical Analysis

 Statistical analysis was performed using SPSS version 18.0 (SPSS Inc., Chicago, USA). All the data presented as mean ± SD. Pain threshold, mRNA levels of EOP precursors, and opioid receptors were used for ANOVA followed by the Bonferroni post hoc test. The correlation coefficient (Pearson's) was used to examine the relations between pain threshold and levels of M-ENK. Statistical significance was evaluated by determining if the *P* value was equal to or less than 0.05. 

## 3. Results

### 3.1. Pain Threshold Changes Induced by Electroacupuncture

**Figure 1:**
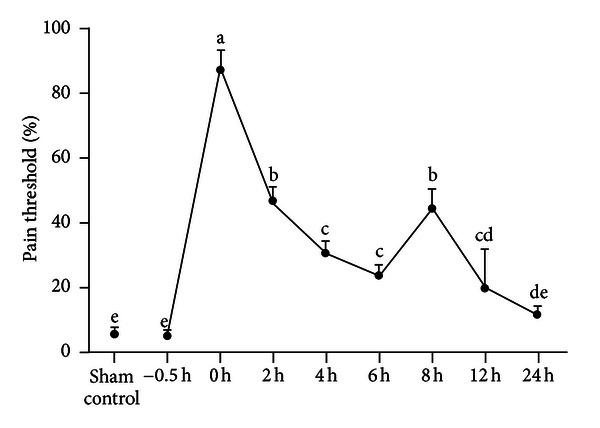
Pain threshold of goats stimulated by EA (mean ± SD, %, *n* = 6). The same letter indicated that no significant difference between pain thresholds at two different time points (*P* > 0.05), and different letters indicated significant difference (*P* < 0.05).

 The analgesic effects of experimental goats were expressed as the pain threshold (Figure 1). The pain threshold of experimental goats at 0.5 h before EA was not different (*P* = 1.00) from that of sham control goats. After EA stimulated the goats, the pain threshold increased and reached the peak at 0 h. Then the pain threshold gradually decreased, began to rebound at 6 h, came to the second peak at 8 h, and then fell gradually again. At 0, 2, 4, 6, 8, and 12 h after EA, the pain threshold increased by 88%, 47%, 32%, 24%, 46%, and 21%, respectively. The pain threshold during the time from 0 to 12 h after EA was higher (*P* < 0.05) than that at 0.5 h before EA. The pain threshold value at 8 h was higher (*P* < 0.01) than that at 4 h, 6 h, 12 h, or 24 h although it was lower (*P* < 0.01) than that at 0 h after EA. There was no difference (*P* = 1.00) between pain thresholds at 2 h and 8 h. 

### 3.2. mRNA Levels of Endogenous Opioid Peptide Precursors in the CNS of Goats

PENK, POMC, and PDYN are the precursors of enkephalin, endorphin, and dynorphin, respectively. The mRNA levels of three endogenous opioid peptide precursors were measured in the analgesia- and distribution-related nuclei or areas. These nuclei or areas mainly included nucleus accumbens (ACB), caudate nucleus (CAU), amygdala (AMY), supraoptic nucleus (SON), paraventricular nucleus of hypothalamus (PVH), ventromedial nucleus of hypothalamus (VMH), arcuate nucleus (ARC), paraventricular nucleus of thalamus (PVT), periaqueductal gray (PAG), dorsal raphe nucleus (DR), habenular nucleus (HB), parabrachial nucleus (PBN), nucleus raphe magnus (NRM), gigantocellular reticular nucleus (GI), solitary nucleus (SOL), neurohypophysis (NH), and spinal cord dorsal horn (SCD). 

The mRNA levels of PENK changed in the measured nuclei or areas in a similar pattern after EA stimulated the animals; they increased (*P* < 0.01) with the peak at 6 h in ACB, PAG, DR, NH, CAU, VMH, PBN, SOL, and PVH, at 8 h in GI, at 12 h in AMY, or at 24 h in HB (Table 2). PENK mRNAs remained higher levels (*P* < 0.05) at 24 h after EA than at 0.5 h before EA in the measured nuclei or areas except ACB. The nucleus or area sequence of the amplitude by which PENK mRNAs increased were NH > PAG > DR = VMH > GI > PVH > PBN > SOL > AMY > CAU > ACB > HB. 

 The mRNA levels of POMC increased (*P* < 0.01) with one or two peaks in the measured nuclei or areas after EA (Table 3). The peak of POMC mRNA levels occurred at 2 h in SOL, at 4 h in CAU, PVH, VMH, and PAG, or at 6 h in NH. There were two peaks of POMC mRNA levels which appeared at 2 h and 8 h in ARC and GI, or at 4 h and 12 h in NRM, or at 4 h and 8 h in AMY, respectively. POMC mRNA levels remained higher (*P* < 0.05) at 24 h after EA than at 0.5 h before EA in the measured nuclei or areas.

The mRNA levels of PDYN began to increase (*P* < 0.05) in most of the measured nuclei or areas at 2 h or 4 h (Table 4). They reached the first peaks at 4 h in AMY, PVT, PAG, PBN, SCD, and CAU or at 6 h in PVH, VMH, SON, and SOL. The second peaks of PDYN mRNA levels, occurred at 12 h, were higher (*P* < 0.05) than the first peaks in all the measured nuclei or areas. PDYN mRNA levels at 12 h increased by times from 0.96 to 2.21, compared with those at 0.5 h before EA stimulated the animals. PDYN mRNAs in AMY, PAG, SCD, and SON remained higher levels (*P* < 0.05) at 24 h while mRNA levels of PDYN in PVT, PBN, PVH, VMH, SOL, and CAU returned to the preacupuncture level (*P* > 0.05).

There were no differences (*P* > 0.05) in mRNA levels of PENK, POMC, or PDYN between sham control goats and experimental goats at 0.5 h before EA in the measured nuclei and areas. 

### 3.3. mRNA Levels of Endogenous Opioid Receptors in the CNS of Goats

 The mRNA levels of *δ*-, *μ*-, or *κ*-receptor were measured in the corresponding nuclei or areas where their ligands are distributed (Tables 5, 6, and 7). The mRNA levels of *δ*-receptor increased (*P* < 0.01) with the peak at 6 h in ACB, PAG, DR, NH, CAU, PVH, VMH, PBN, and SOL, at 8 h in GI, or at 12 h in AMY. *δ*-receptor mRNA remained higher levels in the measured nuclei or areas except ACB at 24 h and kept uptrend in NH, VMH, PBN, SOL, and HB. The *δ*-receptor mRNA levels at the peak in NH, PAG, DR, GI, VMH, PVH, PBN, SOL, AMY, HB, ACB, and CAU increased by 3.24, 2.30, 1.44, 1.36, 1.24, 1.22, 1.20, 1.10, 1.10, 0.96, 0.71, and 0.71 times, respectively. 

The mRNA levels of *μ*-receptor increased (*P* < 0.05) at 0 h after EA and then fluctuated with one or two apparent peaks in the measured nuclei or areas. There was a single peak of *μ*-receptor mRNA levels which occurred at 4 h in VMH and PAG or at 6 h in NH. There were two peaks of *μ*-receptor mRNA levels which appeared at 2 h and 8 h in ARC, GI, and SOL, at 4 h and 8 h in PVH, CAU, and AMY, or at 4 h and 12 h in NRM, respectively. *μ*-receptor mRNA kept higher levels (*P* < 0.05) at 24 h after EA than at 0.5 h before EA in all the measured nuclei or areas. 

The mRNA levels of *κ*-receptor increased (*P* < 0.05) at 0 h, slightly decreased at 8 h, then increased quickly, and reached the peak at 12 h after EA was terminated. Thereafter, *κ*-receptor mRNA levels declined again, but remained higher levels (*P* < 0.05) at 24 h, compared with those at 0.5 h before EA. The *κ*-receptor mRNA levels at 12 h in SCD, PAG, PBN, AMY, PVH, VMH, SON, SOL, PVT, and CAU increased by 1.99, 1.92, 1.67, 1.59, 1.51, 1.43, 1.42, 1.40, 1.31, and 0.81 times, respectively.

There were no differences (*P* > 0.05) in mRNA levels of *δ*-, *μ*-, or *κ*-receptor between sham control goats and experimental goats at 0.5 h before EA in the measured nuclei and areas. 

### 3.4. Levels of Met-Enkephalin in the CNS of Goats

Electroacupuncture induced M-ENK immunoactivities to increase (*P* < 0.05), to reach the peak at 0 h, and then to fall down gradually to the lowest levels at 4–6 h (but still higher than those at 0.5 h before EA) in most measured nuclei or areas. M-ENK immunoactivities rebounded at 4–6 h and came to the second peak at 6–8 h in SOL, VMH, CAU, PBN, AMY, and PVH. At the end of the experiment, M-ENK immunoactivities remained higher levels in the measured nuclei or areas. M-ENK immunoactivities positively correlated (*P* < 0.01) with the pain threshold in the measured nuclei and areas (Table 8). There were no differences (*P* > 0.05) in M-ENK immunoactivities between sham control goats and experimental goats at 0.5 h before EA in the measured nuclei and areas.

## 4. Discussion

### 4.1. The Aftereffect Phenomenon of Analgesia Induced by EA

In the 1970s, researchers used potassium iontophoresis method to quantitatively assess acupuncture-induced change in pain threshold and found that acupuncture caused a gradual increase and slow return in pain threshold [20, 21]. The analgesia during acupuncture is usually believed to be “immediate analgesia” whereas the analgesia which lasts after acupuncture discontinuation is called “analgesic aftereffect of acupuncture.” Some researchers have paid more attention to this aftereffect because they realize it is an important basis for the treatment of pain diseases. Reports showed that hand acupuncture at 5 Hz at human “Hegu” point produced an increase in pain threshold with a peak occurring 20 to 40 min after the needle insertion, and that the threshold returned to the preacupuncture level about 45 min after the needle was removed [22]. Liang et al. [23] alternatively used 20 Hz and 100 Hz of electroacupuncture to stimulate “Kunlun” acupoint of rats with artificial acute arthritis and found the analgesic effect in the inflammatory region lasted for over 60 min after EA was terminated. Zhao et al. [24] applied alternatively 15 Hz and 100 Hz of EA to “Kunlun” and “Xuanzhong” acupoints of rats with adjuvant arthritis and showed that the analgesic aftereffect lasted for about 12 h. These discrepancies in the duration of acupuncture analgesic aftereffect might be caused by different acupuncture methods (electroacupuncture or manual acupuncture), subjects (human or animal), acupoints, acupuncture parameters (frequencies), and so forth. 

Studies show that the analgesic effect induced by EA in goats (a ruminant) is superior to that in rats or human [13, 14]. Therefore, ruminants should be optimal model animals for researches on the mechanisms of EA-induced analgesia (including analgesic aftereffect). Analgesia induced by EA varies in frequency. Our previous study showed that 60 Hz is suitable for the induction of analgesia in goats. In addition, proper prescription of specific acupoints also influences EA-induced analgesic effect. Numerous studies proved that EA at a set of Baihui, Santai, Ergen, and Sanyangluo acupoints elicited an effective analgesia in cattle and goats [13, 16, 25]. In the present study, we used EA of 60 Hz to stimulate goats at a set of acupoints above and found the analgesic effect lasted for 12 h at least, which is in accordance with Zhao's [24] results in rats. However, we also found the pain threshold did not continue to fall back toward the preacupuncture level, but gradually rose to form an apparent peak once again during the time from 6 to 8 h after EA was terminated. This interesting phenomenon has not been reported in human and small experimental animals. The possible explanation for it is species variation. 

### 4.2. Effects of the Gene Expression of EOPs on the EA-Induced Analgesic Aftereffect

Acupuncture practitioners always try to persue a lasting and effective analgesic aftereffect for the treatment of painful diseases because it is a major foundation for optimizing times, interval and session of acupuncture. However, the mechanisms of analgesic aftereffect underlying EA have not been completely elucidated yet. According to the phenomenon that the pain threshold gradually returns to the normal level after acupuncture discontinuation, the analgesic aftereffect should be associated with the change in some physiological substances. Numerous studies confirmed that EOPs in the CNS, mainly M-ENK, *β*-EP, and DYN, played an important role in EA; released EOPs induced by EA acted on their corresponding receptors and exerted immediate analgesic effect [6–9]. Our experiment showed the immunoactivities of M-ENK, a typical representative of EOPs, correlated with the pain threshold after EA was terminated, which indicated that EOPs did participate in the EA-induced analgesic aftereffect. Studies showed that EOPs caused the posttetanic potentiation on the presynaptic nerve terminals of neurons (at least in arcuate nucleus), which led to the lasting synaptic transmission between afferent and efferent pathways [26]. However, these evidences above cannot give a complete explanation for the lasting aftereffect (in some cases even over 24 h) because the released EOPs are enzymolyzed quickly after they combine with their receptors. Therefore, whether EOP gene expression is initiated to replenish the consuming EOPs in EA-induced analgesic aftereffect or not is worthy to be investigated. 

Some studies were involved in gene expressions of EOP precursors after EA treatment. Cui [10] found EA-induced PENK mRNA to express increasingly in the spinal cord and medulla oblongata of rats. The study of Chen et al. [27] indicated that EA elicited POMC mRNA to increase in hypothalamus of rats. Guo et al. [12] determined the levels of PENK and PDYN mRNA at 24 h after EA in the nuclei of hypothalamus and medulla oblongata of rats and found that EA caused PENK mRNA levels to increase in SON, ARC, PVH, VMH, NRM, GI, and PDYN mRNA levels to increase in SON, PVH, VMH, and PBN. These researches above determined EOP precursor mRNAs in a few local districts (nuclei) or at single time point, but did not exhibit their dynamic processes in all analgesia-related nuclei and areas of the CNS.

In this study, dynamic processes of mRNA levels of three EOP precursors (PENK, POMC, and PDYN) were determined in nuclei and areas which were related to analgesic regulation or EOP distribution in the CNS. The results indicated that mRNA levels of the EOP precursors gradually increased, reached the peak during the time from 4 h to 6 h, and remained higher (*P* < 0.05) at 24 h after EA was discontinued in most measured nuclei or areas. In addition, PDYN mRNA levels formed the second peak at about 12 h. Because EOP precursors are translated from their mRNA and then slipped into corresponding EOPs by specific enzymes, mRNA levels of EOP precursors do not completely represent the levels of EOPs. In our previous study, EA at its suitable frequency induced the simultaneous release of the three opioid peptides in extensive analgesia-related nuclei and areas in the CNS of goats [16]. In this study, M-ENK was chosen as a representative of EOPs to investigate the relationships between levels of EOPs and mRNA levels of EOP precursors or pain threshold in the whole experiment. M-ENK levels in the measured nuclei or areas positively correlated (*P* < 0.01) with the pain threshold. The peak of M-ENK levels (at 8 h) lagged behind that of PENK mRNA (at 6 h). The peak (at 8 h) of the pain threshold also lagged behind that (at 4–6 h) of each EOP mRNAs. These results suggested that the initiation of gene expression of the three precursors contributed to the change in pain threshold after acupuncture termination, that is, the analgesic aftereffect. 

The role of DYN-A in EA-induced analgesia in the brain is controversial. Han and Xie [28] found that DYN-A did not produce EA-induced analgesia when it was microinjected into the cerebral ventricle of rats. But Zhang et al. [29] made an opposite conclusion with DYN-A microinjection in rats. Our previous study showed that EA induced DYN-A to increase in most analgesia-related nuclei in the CNS of goats [16]. In this study, there were two peaks of PDYN mRNA levels, one occurred at 4 to 6 h and another at 12 h with the former being lower than the latter. Considering the controversy about the role of DYN in the brain, the gene expression of PENK or POMC may play greater role than that of PDYN in maintaining the analgesic aftereffect. The meanings of the reincrease in PDYN mRNA after 8 h in the analgesic aftereffect need to be investigated. 

Guo et al. [12] used in situ hybridization method to determine the level of POMC mRNA in ARC at 24 h after rats were stimulated by EA of 2 Hz or 100 Hz at “Zusanli” (St.36) and “Sanyinjiao” (SP.6) and found no change in POMC mRNA. However, our results showed that 60 Hz of EA induced POMC mRNA to increase significantly in goats' ARC at 24 h after EA was terminated. This difference may be caused by different frequencies, acupoints, or species. In addition, research methods may be also a contributor. Quantitative real-time PCR used in this study is believed to be more sensitive and reliable than the semiquantitative mRNA methods such as in situ hybridization, dot blot, and northern blot. 

### 4.3. Effects of the Gene Expression of Opioid Receptors on the EA-Induced Analgesic Aftereffect

*δ*-receptor, *μ*-receptor, and *κ*-receptors are three important opioid receptors in the CNS. The affinity of ENK and *β*-EP with *δ*-receptor is almost equal to that of them with *μ*-receptor [30]. DYN has higher affinity with *κ*-receptor than with *δ*- or *μ*-receptor. There are a few reports about the regulation of opioid receptors in EA-induced analgesia. Sun and Han [9] found that EA-induced immediate analgesia is mediated by M-ENK via *δ*-receptor, *β*-EP via *δ*- and *μ*-receptor, and DYN via *κ*-receptor. Some studies showed that the mRNA levels of the three opioid receptors increased at the end of EA [31–33]. However, the dynamic process of opioid receptor mRNAs in EA-induced analgesic aftereffect is not clear yet. In the present study, mRNA levels of the three opioid receptors changed in a similar pattern as those of their ligands; they began to increase at 0 h, reached the peak at 4–6 h or 12 h, and remained higher at 24 h after EA was terminated. The results suggested that EA-induced upregulation of opioid receptor genes may play a role in the analgesic aftereffect. More work on this respect is worthy to be done. 

## 5. Conclusion

 The pain threshold during the time from 0 to 12 h after EA discontinuation was significantly higher than that before acupuncture, which showed that the EA-induced analgesic aftereffect lasted for at least 12 h in goats. The mRNA levels of three EOP precursors (PENK, POMC, and PDYN) and three opioid receptors (*δ*-, *μ*-, and *κ*-receptor) in most analgesia-related nuclei and areas began to increase at 0 h, reached peaks during the time from 4 h to 6 h or at 12 h, and remained higher at 24 h after EA was discontinued in goats. These results, along with the relationships between the dynamic processes of pain threshold and M-ENK (a representative of EOPs) level or EOP precursor mRNA levels, suggested that the initiation of gene expression of the endogenous opioid peptides and their receptors may contribute to the regulation of EA-induced analgesic aftereffect. 

## Figures and Tables

**Table 1:** The quantitative real-time PCR primers of different genes.

Gene	Upstream primer	Downstream primer	Length of PCR product	Referenced GenBank no.
*β*-Actin	5′-ACACGGTGCCCATCTACGA-3′	5′-CCTTGATGTCACGGACGATTT-3′	159 bp	AF481159
PENK	5′-GGCGACCGTGAGGGCAGAATG-3′	5′-GCAGGTTTCCCAGGTCTTGAG-3′	139 bp	NM174141
POMC	5′-GGCGGCCGAGAAGAAGGAC-3′	5′-CTTGATGATGGCGTTTTTGA-3′	148 bp	NM001009266
PDYN	5′-CTTTCCTCACTCCCTTCACCG-3′	5′-TCTCCACCAGGCTGCTACTCA-3′	186 bp	NM174139
*δ*-receptor	5′-TGCTCTCCATTGACTACTACA-3′	5′-AAGAGGAACACGGCAGATTTTG-3′	289 bp	NM001191148
*μ*-receptor	5′-AGTGTGTTATGGGCTGATGA-3′	5′-GCGTCCAGCAGACAATGAACACAG-3′	137 bp	AF266480
*κ*-receptor	5′-CGTCTTCGTCGTCTGCTGG-3′	5′-CAAGGAAGGCGTAGAGAATGG-3′	158 bp	DQ065757

**Table 2:** Levels of PENK gene expression in the CNS of goats stimulated by EA (2^−ΔΔCt^, mean ± SD, *n* = 6).

Nuclei and areas	Sham control	−0.5 h	0 h	2 h	4 h	6 h	8 h	12 h	24 h
ACB	1.06 ± 0.05^d^	1.00 ± 0.00^d^	1.15 ± 0.12^cd^	1.29 ± 0.18^cd^	1.68 ± 0.21^b^	2.01 ± 0.24^a^	1.43 ± 0.17^bc^	1.17 ± 0.16^cd^	1.23 ± 0.14^cd^
CAU	1.03 ± 0.02^d^	1.00 ± 0.00^d^	1.11 ± 0.10^cd^	1.26 ± 0.26^bcd^	1.75 ± 0.21^ab^	2.04 ± 0.19^a^	1.26 ± 0.31^bcd^	1.49 ± 0.35^bcd^	1.54 ± 0.48^abc^
AMY	1.17 ± 0.08^e^	1.00 ± 0.00^e^	1.35 ± 0.19^cd^	1.41 ± 0.11^cd^	1.57 ± 0.13^bc^	1.31 ± 0.12^d^	1.74 ± 0.06^b^	2.10 ± 0.10^a^	1.51 ± 0.13^cd^
PVH	1.06 ± 0.04^e^	1.00 ± 0.00^e^	1.31 ± 0.09^d^	1.38 ± 0.11^cd^	1.89 ± 0.12^b^	2.34 ± 0.10^a^	1.57 ± 0.13^c^	2.06 ± 0.19^b^	1.53 ± 0.15^cd^
VMH	1.24 ± 0.16^e^	1.00 ± 0.00^e^	1.29 ± 0.06^de^	1.47 ± 0.10^cd^	1.69 ± 0.17^bc^	2.42 ± 0.18^a^	1.44 ± 0.21^cd^	1.63 ± 0.15^bc^	1.87 ± 0.20^b^
PAG	1.22 ± 0.21^f^	1.00 ± 0.00^f^	1.64 ± 0.15^ef^	2.46 ± 0.37^cd^	2.86 ± 0.37^ab^	3.37 ± 0.41^a^	2.68 ± 0.43^bc^	2.02 ± 0.36^de^	2.22 ± 0.33^cde^
DR	1.17 ± 0.08^d^	1.00 ± 0.00^d^	1.37 ± 0.09^cd^	1.55 ± 0.05^bcd^	1.98 ± 0.22^ab^	2.42 ± 0.42^a^	1.93 ± 0.43^ab^	1.61 ± 0.37^bc^	1.75 ± 0.31^bc^
HB	1.05 ± 0.04^f^	1.00 ± 0.00^f^	1.22 ± 0.07^def^	1.29 ± 0.17^cde^	1.54 ± 0.21^bc^	1.16 ± 0.07^ef^	1.48 ± 0.16^bcd^	1.72 ± 0.14^ab^	1.93 ± 0.18^a^
PBN	1.16 ± 0.07^e^	1.00 ± 0.00^e^	1.37 ± 0.10^de^	1.50 ± 0.23^cd^	1.77 ± 0.31^bc^	2.21 ± 0.25^a^	1.44 ± 0.15^cd^	1.67 ± 0.21^bcd^	1.89 ± 0.19^ab^
GI	1.05 ± 0.09^e^	1.00 ± 0.00^e^	1.27 ± 0.06^d^	1.39 ± 0.17^cd^	1.61 ± 0.12^c^	1.93 ± 0.18^b^	2.39 ± 0.20^a^	1.59 ± 0.11^c^	1.33 ± 0.11^d^
SOL	1.08 ± 0.12^e^	1.00 ± 0.00^e^	1.33 ± 0.11^d^	1.49 ± 0.21^bcd^	1.71 ± 0.21^b^	2.12 ± 0.16^a^	1.36 ± 0.13^cd^	1.52 ± 0.17^bcd^	1.65 ± 0.22^bc^
NH	1.16 ± 0.04^e^	1.00 ± 0.00^e^	1.54 ± 0.13^d^	2.17 ± 0.22^c^	3.22 ± 0.13^b^	4.27 ± 0.43^a^	2.19 ± 0.37^c^	1.59 ± 0.24^d^	2.17 ± 0.23^c^

There was difference (*P* < 0.05) between the values with different letters, and no difference (*P* > 0.05) with the same letters in a line. The letters in the following tables have the same meanings.

**Table 3:** Levels of POMC gene expression in the CNS of goats stimulated by EA (2^−ΔΔCt^, mean ± SD, *n* = 6).

Nuclei and areas	Sham control	−0.5 h	0 h	2 h	4 h	6 h	8 h	12 h	24 h
CAU	1.03 ± 0.02^c^	1.00 ± 0.00^c^	1.25 ± 0.09^bc^	1.38 ± 0.16^b^	2.03 ± 0.23^a^	1.37 ± 0.14^b^	1.81 ± 0.16^a^	1.27 ± 0.05^b^	1.46 ± 0.13^b^
AMY	1.09 ± 0.05^d^	1.00 ± 0.00^d^	1.28 ± 0.08^cd^	1.34 ± 0.08^bc^	1.64 ± 0.23^ab^	1.39 ± 0.22^abc^	1.69 ± 0.30^a^	1.25 ± 0.06^cd^	1.39 ± 0.10^abc^
PVH	1.13 ± 0.03^e^	1.00 ± 0.00^e^	1.54 ± 0.12^cd^	1.96 ± 0.20^ab^	2.31 ± 0.17^a^	1.39 ± 0.25^d^	1.84 ± 0.14^bc^	1.51 ± 0.12^cd^	1.49 ± 0.36^cd^
VMH	1.10 ± 0.06^f^	1.00 ± 0.00^f^	1.17 ± 0.07^ef^	1.31 ± 0.13^e^	2.36 ± 0.10^a^	1.72 ± 0.16^bc^	1.86 ± 0.15^b^	1.35 ± 0.11^de^	1.57 ± 0.13^cd^
ARC	1.13 ± 0.03^e^	1.00 ± 0.00^e^	1.36 ± 0.13^d^	2.17 ± 0.12^b^	1.54 ± 0.14^cd^	2.24 ± 0.16^b^	2.76 ± 0.21^a^	2.08 ± 0.13^b^	1.78 ± 0.14^c^
PAG	1.06 ± 0.03^d^	1.00 ± 0.00^d^	1.41 ± 0.10^c^	1.66 ± 0.21^bc^	2.56 ± 0.16^a^	1.84 ± 0.10^b^	1.66 ± 0.17^bc^	1.43 ± 0.20^c^	1.64 ± 0.22^bc^
NRM	1.06 ± 0.04^d^	1.00 ± 0.00^d^	1.42 ± 0.11^c^	1.92 ± 0.14^b^	2.54 ± 0.18^a^	1.61 ± 0.25^bc^	1.44 ± 0.27^c^	2.32 ± 0.20^a^	1.47 ± 0.14^c^
GI	1.13 ± 0.03^e^	1.00 ± 0.00^e^	1.57 ± 0.09^d^	2.61 ± 0.19^a^	1.93 ± 0.17^bc^	2.14 ± 0.27^b^	2.65 ± 0.19^a^	1.81 ± 0.15^bcd^	1.67 ± 0.20^cd^
SOL	1.05 ± 0.07^c^	1.00 ± 0.00^c^	1.47 ± 0.09^b^	2.12 ± 0.17^a^	1.79 ± 0.18^ab^	1.61 ± 0.19^b^	1.93 ± 0.36^ab^	1.70 ± 0.48^ab^	1.83 ± 0.13^ab^
NH	1.07 ± 0.03^e^	1.00 ± 0.00^e^	1.33 ± 0.12^d^	1.42 ± 0.18^d^	1.96 ± 0.14^c^	2.81 ± 0.08^a^	1.75 ± 0.08^c^	1.43 ± 0.06^d^	2.24 ± 0.19^b^

**Table 4:** Levels of PDYN gene expression in the CNS of goats stimulated by EA (2^−ΔΔCt^, mean ± SD, *n* = 6).

Nuclei and areas	Sham control	−0.5 h	0 h	2 h	4 h	6 h	8 h	12 h	24 h
CAU	1.08 ± 0.03^de^	1.00 ± 0.00^de^	1.05 ± 0.09^de^	0.87 ± 0.03^e^	1.68 ± 0.09^b^	1.36 ± 0.07^c^	1.07 ± 0.02^d^	1.96 ± 0.08^a^	1.12 ± 0.07^d^
AMY	1.17 ± 0.04^e^	1.00 ± 0.00^e^	1.30 ± 0.08^d^	1.53 ± 0.06^c^	1.87 ± 0.09^b^	1.30 ± 0.03^d^	1.40 ± 0.05^cd^	2.75 ± 0.12^a^	1.40 ± 0.08^cd^
PVH	1.06 ± 0.02^c^	1.00 ± 0.00^c^	1.23 ± 0.12^c^	1.29 ± 0.09^c^	1.46 ± 0.11^c^	1.82 ± 0.09^b^	1.25 ± 0.13^c^	2.86 ± 0.24^a^	1.16 ± 0.04^c^
VMH	1.09 ± 0.04^d^	1.00 ± 0.00^d^	1.10 ± 0.07^d^	1.17 ± 0.11^cd^	1.45 ± 0.13^c^	1.88 ± 0.11^b^	1.10 ± 0.07^d^	2.57 ± 0.16^a^	1.18 ± 0.08^cd^
SON	1.10 ± 0.03^e^	1.00 ± 0.00^e^	1.15 ± 0.13^de^	1.22 ± 0.09^de^	1.61 ± 0.07^bc^	1.84 ± 0.07^b^	1.35 ± 0.12^cd^	2.53 ± 0.14^a^	1.37 ± 0.11^cd^
PVT	1.20 ± 0.08^b^	1.00 ± 0.00^b^	1.22 ± 0.10^b^	1.27 ± 0.15^b^	2.11 ± 0.18^a^	1.41 ± 0.16^b^	1.23 ± 0.14^b^	2.40 ± 0.25^a^	1.21 ± 0.11^b^
PAG	1.15 ± 0.05^e^	1.00 ± 0.00^e^	1.21 ± 0.11^de^	1.59 ± 0.11^c^	1.89 ± 0.07^b^	1.51 ± 0.13^cd^	1.25 ± 0.11^de^	3.11 ± 0.09^a^	1.36 ± 0.09^cd^
PBN	1.07 ± 0.03^d^	1.00 ± 0.00^d^	1.27 ± 0.09^cd^	1.31 ± 0.14^c^	1.66 ± 0.09^b^	1.26 ± 0.07^cd^	1.08 ± 0.04^cd^	2.78 ± 0.17^a^	1.23 ± 0.11^cd^
SOL	1.11 ± 0.04^c^	1.00 ± 0.00^c^	1.37 ± 0.09^bc^	1.43 ± 0.09^bc^	1.48 ± 0.20^b^	1.49 ± 0.16^b^	1.15 ± 0.11^bc^	2.44 ± 0.23^a^	1.27 ± 0.15^bc^
SCD	1.07 ± 0.02^e^	1.00 ± 0.00^e^	1.33 ± 0.12^cd^	1.30 ± 0.06^cd^	1.63 ± 0.10^b^	1.39 ± 0.03^c^	1.14 ± 0.04^de^	3.21 ± 0.15^a^	1.52 ± 0.09^bc^

**Table 5:** Levels of *δ*-receptor gene expression in the CNS of goats stimulated by EA (2^−ΔΔCt^, mean ± SD, *n* = 6).

Nuclei and areas	Sham control	−0.5 h	0 h	2 h	4 h	6 h	8 h	12 h	24 h
ACB	1.05 ± 0.03^d^	1.00 ± 0.00^d^	1.05 ± 0.06^cd^	1.19 ± 0.07^c^	1.39 ± 0.11^b^	1.79 ± 0.09^a^	1.52 ± 0.10^b^	1.15 ± 0.13^cd^	1.10 ± 0.08^cd^
CAU	1.08 ± 0.04^de^	1.00 ± 0.00^de^	0.99 ± 0.05^e^	1.10 ± 0.05^de^	1.45 ± 0.09^b^	1.71 ± 0.12^a^	1.16 ± 0.12^cd^	1.32 ± 0.10^bc^	1.29 ± 0.10^bc^
AMY	1.05 ± 0.04^f^	1.00 ± 0.00^f^	1.23 ± 0.09^e^	1.43 ± 0.09^cd^	1.59 ± 0.10^bc^	1.32 ± 0.09^de^	1.76 ± 0.09^b^	2.10 ± 0.13^a^	1.48 ± 0.12^cd^
PVH	1.08 ± 0.09^f^	1.00 ± 0.00^f^	1.25 ± 0.08^e^	1.36 ± 0.10^de^	1.83 ± 0.10^b^	2.22 ± 0.12^a^	1.56 ± 0.10^c^	2.06 ± 0.17^a^	1.49 ± 0.09^cd^
VMH	1.12 ± 0.05^f^	1.00 ± 0.00^f^	1.30 ± 0.07^e^	1.44 ± 0.10^de^	1.69 ± 0.13^bc^	2.24 ± 0.11^a^	1.44 ± 0.12^de^	1.63 ± 0.09^cd^	1.87 ± 0.17^b^
PAG	1.23 ± 0.16^f^	1.00 ± 0.00^f^	1.64 ± 0.09^e^	2.41 ± 0.19^cd^	2.79 ± 0.15^b^	3.30 ± 0.21^a^	2.49 ± 0.17^bc^	2.11 ± 0.21^d^	2.16 ± 0.19^d^
DR	1.13 ± 0.07^f^	1.00 ± 0.00^f^	1.29 ± 0.08^ef^	1.51 ± 0.08^de^	1.89 ± 0.16^bc^	2.44 ± 0.31^a^	1.96 ± 0.15^b^	1.63 ± 0.10^cd^	1.69 ± 0.15^bcd^
HB	0.99 ± 0.06^f^	1.00 ± 0.00^f^	1.19 ± 0.08^e^	1.29 ± 0.11^de^	1.55 ± 0.11^bc^	1.18 ± 0.08^ef^	1.48 ± 0.09^cd^	1.71 ± 0.10^b^	1.96 ± 0.15^a^
PBN	1.07 ± 0.04^f^	1.00 ± 0.00^f^	1.37 ± 0.11^e^	1.42 ± 0.13^e^	1.75 ± 0.13^bc^	2.20 ± 0.10^a^	1.49 ± 0.08^de^	1.65 ± 0.12^cd^	1.85 ± 0.07^b^
GI	1.04 ± 0.05^e^	1.00 ± 0.00^e^	1.25 ± 0.08^d^	1.35 ± 0.14^d^	1.61 ± 0.08^c^	1.90 ± 0.15^b^	2.36 ± 0.12^a^	1.59 ± 0.08^c^	1.30 ± 0.07^d^
SOL	1.07 ± 0.03^f^	1.00 ± 0.00^f^	1.33 ± 0.08^e^	1.49 ± 0.10^de^	1.74 ± 0.09^b^	2.10 ± 0.10^a^	1.37 ± 0.12^e^	1.55 ± 0.10^cd^	1.69 ± 0.09^bc^
NH	1.10 ± 0.05^e^	1.00 ± 0.00^e^	1.57 ± 0.09^d^	2.24 ± 0.10^c^	3.24 ± 0.13^b^	4.24 ± 0.14^a^	2.34 ± 0.11^c^	1.61 ± 0.12^d^	2.17 ± 0.14^c^

**Table 6:** Levels of *μ*-receptor gene expression in the CNS of goats stimulated by EA (2^−ΔΔCt^, mean ± SD, *n* = 6).

Nuclei and areas	Sham control	−0.5 h	0 h	2 h	4 h	6 h	8 h	12 h	24 h
CAU	1.05 ± 0.03^d^	1.00 ± 0.00^d^	1.26 ± 0.11^c^	1.38 ± 0.15^bc^	1.99 ± 0.10^a^	1.35 ± 0.14^bc^	1.81 ± 0.13^a^	1.29 ± 0.10^bc^	1.48 ± 0.12^b^
AMY	1.04 ± 0.04^c^	1.00 ± 0.00^c^	1.26 ± 0.11^b^	1.39 ± 0.10^b^	1.53 ± 0.14^a^	1.38 ± 0.08^b^	1.64 ± 0.15^a^	1.26 ± 0.08^b^	1.38 ± 0.09^b^
PVH	1.11 ± 0.04^e^	1.00 ± 0.00^e^	1.48 ± 0.10^d^	2.01 ± 0.19^b^	2.26 ± 0.13^a^	1.36 ± 0.08^d^	1.74 ± 0.13^c^	1.47 ± 0.11^d^	1.49 ± 0.10^d^
VMH	1.12 ± 0.06^e^	1.00 ± 0.00^e^	1.16 ± 0.11^de^	1.38 ± 0.10^cd^	2.24 ± 0.12^a^	1.75 ± 0.11^b^	1.79 ± 0.13^b^	1.39 ± 0.15^c^	1.46 ± 0.12^c^
ARC	1.08 ± 0.04^e^	1.00 ± 0.00^e^	1.36 ± 0.16^d^	2.08 ± 0.15^b^	1.50 ± 0.12^cd^	2.20 ± 0.12^b^	2.82 ± 0.18^a^	2.10 ± 0.13^b^	1.71 ± 0.10^c^
PAG	1.18 ± 0.11^e^	1.00 ± 0.00^e^	1.44 ± 0.12^d^	1.71 ± 0.14^bc^	2.41 ± 0.21^a^	1.72 ± 0.11^b^	1.67 ± 0.11^bcd^	1.45 ± 0.16^cd^	1.54 ± 0.16^bcd^
NRM	1.09 ± 0.08^d^	1.00 ± 0.00^d^	1.44 ± 0.14^c^	1.99 ± 0.16^b^	2.41 ± 0.15^a^	1.58 ± 0.15^c^	1.45 ± 0.13^c^	2.09 ± 0.08^b^	1.51 ± 0.11^c^
GI	1.21 ± 0.15^e^	1.00 ± 0.00^e^	1.51 ± 0.08^d^	2.54 ± 0.15^a^	1.88 ± 0.18^bc^	2.12 ± 0.19^b^	2.47 ± 0.15^a^	1.78 ± 0.14^cd^	1.74 ± 0.16^cd^
SOL	1.08 ± 0.07^e^	1.00 ± 0.00^e^	1.44 ± 0.14^d^	2.09 ± 0.13^a^	1.77 ± 0.10^bc^	1.63 ± 0.12^cd^	2.01 ± 0.28^ab^	1.68 ± 0.21^cd^	1.72 ± 0.16^bcd^
NH	1.08 ± 0.06^e^	1.00 ± 0.00^e^	1.33 ± 0.15^d^	1.38 ± 0.15^cd^	2.02 ± 0.15^b^	2.53 ± 0.13^a^	1.59 ± 0.13^c^	1.47 ± 0.10^cd^	2.23 ± 0.12^b^

**Table 7:** Levels of *κ*-receptor gene expression in the CNS of goats stimulated by EA (2^−ΔΔCt^, mean ± SD, *n* = 6).

Nuclei and areas	Sham control	−0.5 h	0 h	2 h	4 h	6 h	8 h	12 h	24 h
CAU	0.98 ± 0.06^c^	1.00 ± 0.00^c^	1.11 ± 0.06^c^	0.99 ± 0.09^c^	1.55 ± 0.12^b^	1.41 ± 0.08^b^	1.10 ± 0.09^c^	1.81 ± 0.10^a^	1.10 ± 0.10^c^
AMY	1.17 ± 0.13^d^	1.00 ± 0.00^d^	1.33 ± 0.10^c^	1.37 ± 0.13^c^	1.65 ± 0.07^b^	1.33 ± 0.14^c^	1.15 ± 0.09^cd^	2.59 ± 0.18^a^	1.23 ± 0.15^cd^
PVH	1.10 ± 0.04^d^	1.00 ± 0.00^d^	1.28 ± 0.12^c^	1.32 ± 0.09^c^	1.47 ± 0.13^c^	1.80 ± 0.14^b^	1.24 ± 0.09^c^	2.51 ± 0.21^a^	1.24 ± 0.14^cd^
VMH	1.09 ± 0.05^e^	1.00 ± 0.00^e^	1.15 ± 0.11^de^	1.20 ± 0.10^d^	1.44 ± 0.13^c^	1.89 ± 0.10^b^	1.15 ± 0.08^de^	2.43 ± 0.15^a^	1.18 ± 0.09^de^
SON	1.16 ± 0.14^e^	1.00 ± 0.00^e^	1.22 ± 0.11^de^	1.29 ± 0.11^d^	1.58 ± 0.11^bc^	1.72 ± 0.10^b^	1.45 ± 0.13^cd^	2.42 ± 0.22^a^	1.41 ± 0.15^cd^
PVT	1.06 ± 0.03^d^	1.00 ± 0.00^d^	1.26 ± 0.08^c^	1.36 ± 0.08^c^	2.01 ± 0.10^b^	1.42 ± 0.11^c^	1.25 ± 0.13^c^	2.31 ± 0.14^a^	1.29 ± 0.10^c^
PAG	1.09 ± 0.13^e^	1.00 ± 0.00^e^	1.25 ± 0.08^d^	1.56 ± 0.10^c^	1.93 ± 0.10^b^	1.58 ± 0.08^c^	1.29 ± 0.08^d^	2.92 ± 0.16^a^	1.41 ± 0.14^cd^
PBN	1.07 ± 0.06^e^	1.00 ± 0.00^e^	1.31 ± 0.12^cd^	1.35 ± 0.12^c^	1.67 ± 0.09^b^	1.27 ± 0.06^cd^	1.13 ± 0.11^de^	2.67 ± 0.15^a^	1.37 ± 0.16^c^
SOL	1.11 ± 0.15^d^	1.00 ± 0.00^d^	1.37 ± 0.12^bc^	1.45 ± 0.11^b^	1.51 ± 0.13^b^	1.53 ± 0.11^b^	1.16 ± 0.11^cd^	2.40 ± 0.14^a^	1.31 ± 0.13^bc^
SCD	1.06 ± 0.04^e^	1.00 ± 0.00^e^	1.38 ± 0.10^cd^	1.35 ± 0.10^cd^	1.59 ± 0.11^b^	1.42 ± 0.07^bcd^	1.23 ± 0.11^d^	2.99 ± 0.18^a^	1.55 ± 0.12^bc^

**Table 8:** M-ENK immunoactivities in the CNS of goats stimulated by EA (mean ± SD, *n* = 6).

Nuclei and areas	Sham control	−0.5 h	0 h	2 h	4 h	6 h	8 h	12 h	24 h	Correlation with pain threshold (*r*)
ACB	15.96 ± 1.53^c^	16.17 ± 2.03^c^	35.76 ± 4.50^a^	27.78 ± 2.15^b^	23.92 ± 2.03^b^	18.47 ± 2.96^c^	17.55 ± 0.89^c^	17.27 ± 1.91^c^	15.37 ± 1.72^c^	0.874**
CAU	14.19 ± 1.21^d^	13.67 ± 2.88^d^	33.06 ± 3.62^a^	28.69 ± 2.91^ab^	23.48 ± 3.94^bc^	17.72 ± 3.65^cd^	23.02 ± 3.12^bc^	16.21 ± 2.26^d^	14.80 ± 2.02^d^	0.819**
AMY	10.74 ± 1.55^g^	10.62 ± 2.07^g^	47.74 ± 5.94^a^	37.40 ± 3.14^b^	28.69 ± 2.91^cd^	34.41 ± 3.69^bc^	25.13 ± 2.12^de^	18.42 ± 1.05^f^	21.16 ± 1.93^ef^	0.808**
PVH	17.11 ± 2.28^c^	17.68 ± 4.59^c^	43.99 ± 5.78^a^	37.71 ± 2.98^a^	29.18 ± 5.47^b^	21.78 ± 2.14^bc^	28.14 ± 1.40^b^	23.95 ± 4.93^bc^	28.41 ± 1.82^b^	0.729**
VMH	12.14 ± 1.81^e^	11.66 ± 3.21^e^	41.24 ± 2.53^a^	31.65 ± 3.07^b^	29.94 ± 2.72^b^	19.48 ± 1.57^d^	27.05 ± 2.55^bc^	24.85 ± 2.30^c^	18.79 ± 1.29^d^	0.848**
PAG	8.04 ± 0.84^e^	7.91 ± 1.18^e^	32.13 ± 2.13^a^	26.58 ± 1.00^b^	21.04 ± 3.76^c^	15.73 ± 1.69^d^	18.57 ± 1.32^cd^	21.28 ± 1.67^c^	21.70 ± 1.53^c^	0.748**
DR	11.48 ± 1.64^e^	10.92 ± 1.73^e^	28.41 ± 1.82^a^	22.96 ± 1.94^b^	19.32 ± 1.64^bc^	14.48 ± 1.06^de^	16.94 ± 0.79^cd^	20.66 ± 1.46^bc^	18.31 ± 0.72^cd^	0.759**
HB	16.51 ± 1.44^cd^	15.76 ± 1.46^cd^	26.19 ± 1.21^a^	24.17 ± 1.04^a^	19.49 ± 1.75^b^	19.06 ± 1.87^b^	17.87 ± 1.04^bc^	16.75 ± 2.35^bcd^	15.52 ± 1.27^d^	0.837**
PBN	7.79 ± 1.10^e^	6.75 ± 1.24^e^	26.80 ± 3.72^a^	23.44 ± 1.47^a^	19.23 ± 1.62^b^	14.02 ± 1.88^cd^	16.80 ± 1.87^bc^	12.70 ± 1.12^d^	11.16 ± 1.53^d^	0.857**
GI	11.56 ± 1.27^d^	10.51 ± 1.42^d^	19.13 ± 1.33^a^	18.14 ± 1.09^a^	15.03 ± 0.97^bc^	14.60 ± 1.14^bc^	11.70 ± 1.03^d^	12.73 ± 1.47^cd^	15.70 ± 1.27^b^	0.605**
SOL	14.03 ± 1.77^g^	14.44 ± 0.93^g^	49.83 ± 1.15^a^	40.99 ± 2.05^b^	30.82 ± 1.40^c^	24.32 ± 1.69^e^	27.67 ± 1.34^d^	26.48 ± 1.25^de^	19.49 ± 1.38^f^	0.911**

**Means the levels of M-ENK correlate with the pain threshold at the 0.01 level.
